# Case Report: Pulmonary echinococcosis misdiagnosed as bronchogenic pulmonary cysts

**DOI:** 10.3389/fmed.2025.1533124

**Published:** 2025-03-17

**Authors:** Qingcheng Yang, Lyubo Wang, Yuanlong Shi, Siyun Liu, Daoguang Fan, Bencheng Wu, Yi Duan, Chenjun Xin, Lincan Duan

**Affiliations:** ^1^Department of Thoracic Surgery, Yunnan Cancer Hospital, Peking University Cancer Hospital, Kunming, China; ^2^Department of Urology, Yunnan Cancer Hospital, Peking University Cancer Hospital, Kunming, China; ^3^Department of Thoracic Surgery, Pu’er People’s Hospital, Pu’er, China

**Keywords:** pulmonary echinococcosis, bronchogenic pulmonary cysts, diagnose, treatments, misdiagnosis

## Abstract

Echinococcosis, also known as hydatid disease, is a zoonotic parasitic infection that poses a significant risk to human health. This article delineates the diagnostic and therapeutic course of a patient afflicted with pulmonary echinococcosis who was admitted to the Department of Thoracic Surgery II at Yunnan Cancer Hospital in April 2024. The patient exhibited a history of extensive exposure to livestock and a penchant for consuming undercooked meat. Prior to undergoing surgical intervention, the patient was initially diagnosed with bronchogenic pulmonary cyst. However, subsequent pathological examination revealed a diagnosis of pulmonary echinococcosis. The rarity of the disease and the paucity of experience in diagnosis and treatment rendered the patient’s case a valuable opportunity to elucidate the diagnostic and therapeutic journey. This report aims to provide a comprehensive reference for the accurate identification and treatment of pulmonary echinococcosis in future clinical practice.

## Introduction

Echinococcosis, a globally prevalent chronic zoonotic parasitic disease, arises from infection with the larvae of the *Echinococcus* tapeworm (1). This infection leads to the gradual formation of an encysted capsule within the host, hence the alternative designation of the condition as an encysted disease. The disease manifests primarily in two forms: multilocular echinococcosis and *Echinococcus granulosus* infection, with the latter being the more prevalent variant (2). The prevalence of *Echinococcus granulosus* infection is predominantly high in regions such as sub-Saharan Africa, Central Asia, South America, and the Mediterranean area. Additionally, it is highly prevalent among pastoral communities in the western and northwestern parts of China. This infection is classified as one of the 17 neglected tropical diseases by the World Health Organization (WHO) (3). China is among the countries with a high prevalence of *Echinococcus granulosus*, with cases reported across 23 provinces (autonomous regions). The cumulative number of infected individuals exceeds one million. The disease manifests with particularly high incidence in the pastoral regions of Sichuan, Qinghai, Xinjiang, and Inner Mongolia, where its impact is most pronounced (4). In instances of accidental ingestion of food, water, or beverages contaminated with *Echinococcus granulosus* eggs, the eggs undergo hatching within the intestinal tract, resulting in the release of hexacanth larvae. These larvae penetrate the intestinal wall, enter the bloodstream, and eventually reach the liver, lungs, or other organs, where they form cysts (5). *Echinococcus granulosus*, a parasitic flatworm, has a protracted growth cycle within the human body. Its morphology is intricate and variable, typically manifesting as a cystic lesion with clearly delineated boundaries, which may be accompanied by calcification. The absence of specific clinical symptoms in most patients contributes to misdiagnosis, underdiagnosis, and delayed treatment. This poses a significant public health threat (6). Bronchogenic pulmonary cysts are localized cystic lesions of the lung parenchyma due to congenital lung developmental abnormalities. CT images often show smooth, round, or similarly rounded lesions that usually lack specific imaging features, clinical symptoms, and signs (7). Consequently, there is a degree of similarity between the imaging characteristics of certain cases of pulmonary echinococcosis and those of bronchogenic pulmonary cysts. In this regard, we present a case study of a patient admitted to the Department of Thoracic Surgery II at Yunnan Provincial Cancer Hospital in April 2024, with the aim of providing clinical guidance and insights.

## Case data

**Figure 1:**
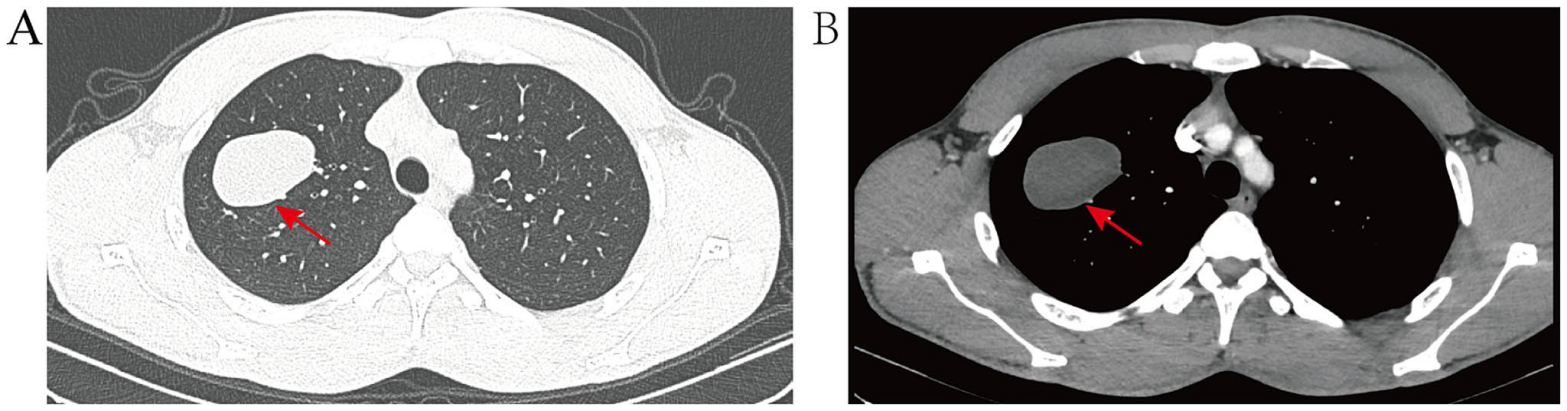
**(A)** Pre-treatment CT lung window image displays the lesion situated in the apical segment of the upper lobe of the right lung, encompassing portions of both the anterior and posterior segments. The lesion presents as an oval-shaped, solid mass with distinct margins. **(B)** Pre-treatment CT mediastinum window image reveals no significant enhancement post-contrast, exhibiting uniform density throughout, with no evidence of calcification.

The patient, a 23-year-old male from Baoshan, Yunnan Province, was admitted to Yunnan Cancer Hospital in April 2024 for a persistent cough, with no obvious clinical symptoms. The patient reported a history of prolonged, frequent contact with cattle and sheep, spanning over 3 years, and engaged in livestock slaughtering for a period exceeding 1 year, primarily involving cattle, sheep, and pigs. He also noted a tendency to consume raw meat and did not recall drinking raw water. The patient was physically fit in the past, with no history of hereditary diseases, no family history of tumors, no history of allergies, and no history of surgery. A chest computed tomography (CT) scan was conducted (Figure 1), revealing an oval-shaped, low-density lesion in the upper lobe of the right lung, measuring approximately 5.0 cm by 3.4 cm. The lesion exhibited well-defined borders and did not demonstrate significant enhancement post-contrast administration. It extended inferiorly into the lumen of the bronchus of the anterior segment. In the surrounding area, speckled foci and small nodular density shadows were observed. Given these findings, a preliminary diagnosis of bronchogenic pulmonary cysts is suggested. The hilar and mediastinal regions of the lungs showed no evidence of lymph node enlargement or other significant findings. Concurrently, comprehensive blood tests, encompassing assessments of liver and kidney function and electrolyte levels, yielded normal results devoid of any remarkable findings.

## Treatment and diagnosis

**Figure 2:**
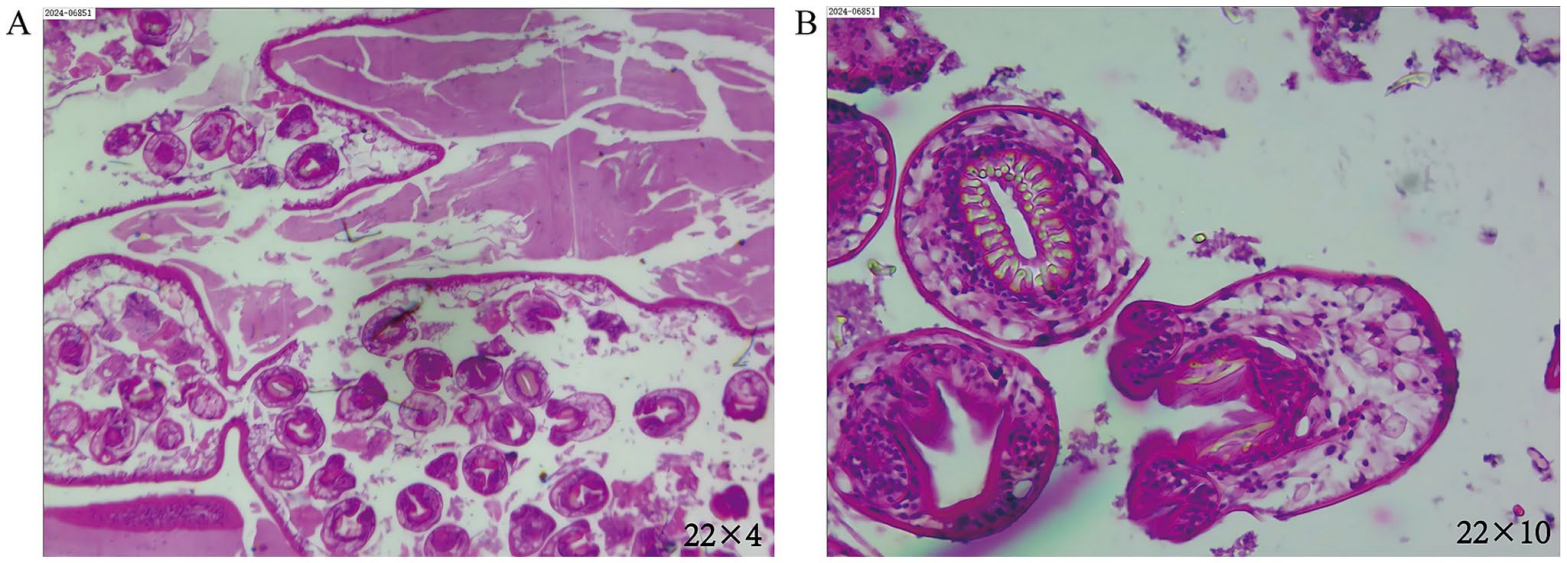
**(A)** The magnification is set at eyepiece 22 multiplied by objective 4. **(B)** The magnification is set at eyepiece 22 multiplied by objective 10. In the illustrations, homogeneous red staining is observed in certain areas of the postoperative lesions, with a scattered distribution of structures resembling *Echinococcus*. Visible are the characteristic hooklets and protoscolices, indicative of *Echinococcus granulosus*.

Following the completion of a comprehensive diagnostic imaging and laboratory evaluation, including chest CT, cranial CT, abdominal ultrasound, systemic lymph node ultrasound, and blood routine, as well as liver and kidney function, electrolytes, and other pertinent examinations and tests, the chest CT revealed the presence of upper lobe abnormalities in the right lung. However, the remaining examinations and tests yielded no additional abnormalities. Based on the characteristics of the CT image, a preliminary diagnosis of a cyst of pulmonary bronchogenic origin was rendered. The patient’s lesions were located in close proximity to small bronchioles, which posed a challenge in their visualization through bronchoscopy. To mitigate the potential risks associated with bronchoscopy, such as infection and bleeding due to cyst rupture, the patient opted against undergoing the procedure. Notwithstanding the absence of any atypical symptoms, the patient underwent a surgical procedure on April 7, 2024, consisting of an apical segmentectomy, accompanied by a partial resection of the anterior and posterior segments of the upper lobe of the right lung. This surgical intervention was deemed necessary due to concerns regarding the tumor’s size and the potential for further growth. Intraoperatively, the right lung exhibited normal overall development, the pleura of the lesion area did not show obvious wrinkles, and the majority of the tumor was located in the area of the apical segment of the upper lobe of the right lung. Following the meticulous separation of the blood vessels and bronchial tubes of the apical segment, a comprehensive resection of the apical segment, in conjunction with a portion of the anterior and posterior segments of the lung, was performed. It is noteworthy that the tumor remained intact throughout the procedure, exhibiting no signs of ulceration. The tissues of the lobes of the lung that had been resected in proximity to the lesion were meticulously packaged into specimen bands and subsequently extracted from the exterior of the body. Intraoperative frozen pathological examination showed benign. Postoperative pathologic examination showed (Figure 2) *Echinococcus granulosus*. He was discharged to the hospital for infectious diseases for further consultation and guidance for follow-up. The patient is currently undergoing regular albendazole treatment and is being closely monitored. A follow-up visit was conducted at our hospital in June 2024, during which the patient exhibited a satisfactory recovery, with no indications of recurrence or adverse effects resulting from the treatment. Thereafter, the patient underwent regular follow-ups at the local hospital, and no significant or abnormal findings were reported in the telephone follow-ups.

## Discussion

Pulmonary echinococcosis is a zoonotic parasitic disease characterized by the larvae’s journey through the bloodstream to the pulmonary capillaries, where they parasitize and induce lesions. The occurrence of this disease in the lungs is second most common after the liver, with a documented human mortality rate of 4%, predominantly attributed to anaphylactic shock resulting from cyst rupture (8). The presence of Echinococcosis-induced occupying sacs or lesions can be identified through a variety of clinical examinations, including radiography, ultrasound, CT, or MRI. Once a suspicion of an occupying sac has been detected, indirect hemagglutination tests or enzyme immunoassays are utilized to confirm the diagnosis. Nevertheless, the gold standard for confirming echinococcosis remains a postoperative pathological examination (9). In the diagnosis of pulmonary echinococcosis, chest imaging plays a pivotal role. The manifestation of simple pulmonary *Echinococcus granulosus* encapsulation is characterized by the presence of one or more distinctly outlined, round or slightly irregular opacities on imaging. The distinctive features that significantly inform the clinical differential diagnosis include the sharp demarcation from the adjacent lung parenchyma, the formation of an acute angle with the pleural line, and the presence of a neutrophilic capsule. As the capsule expands and leads to bronchiolar erosion, air accumulation may occur between the outer and inner sacs, resulting in the manifestation of the “meniscus sign.” Further accumulation of air within the inner sacs can lead to the emergence of the “arch sign.” Rupture of the inner sacs gives rise to an air-fluid interface, with remnants of the inner sacs floating atop the sac fluid, producing the “water lily sign.” This sign is a significant radiographic feature indicative of certain forms of pulmonary parasitic disease (10). Bronchogenic pulmonary cysts are congenital malformations that arise from the abnormal development of the embryonic trachea and bronchi. Their radiographic manifestation on X-rays and CT scans is typified by a round, cystic appearance, with the cysts displaying characteristics of soft tissue. The classification of these cysts is based on their anatomical location and includes three distinct types: mediastinal, intrapulmonary, and ectopic (11). Surgical excision of the cysts has been demonstrated to mitigate the risks associated with combined compression, infection, and hemorrhage of pulmonary cysts. Furthermore, it has been shown to significantly reduce the potential for malignant transformation of the cysts (12).

The CT images of the patient detailed in this case report lacked the characteristic features typically associated with pulmonary echinococcosis, such as “subcapsules” and the “water lily sign.” Consequently, a definitive diagnosis based solely on CT imaging was challenging. The management of echinococcosis is tailored to the stage of disease advancement and the specific location of the lesion. Surgical resection is regarded as the primary treatment for cystic echinococcosis. It is imperative to exercise caution during surgical intervention to avert the rupture of the cyst, as this could precipitate anaphylactic reactions and the potential dissemination of the disease. In the context of pulmonary echinococcosis, the preoperative administration of albendazole is a contentious issue. Some evidence suggests that preoperative administration of albendazole could potentially increase the likelihood of cyst rupture (10). In this case, the patient was not initially diagnosed with pulmonary echinococcosis prior to surgery and thus did not receive any medication preoperatively. Subsequent to discharge, the patient was counseled to seek consultation at the infectious disease hospital to forestall the reemergence of the disease. The patient was subsequently transferred to the infectious disease facility for continued care and is now undergoing regular albendazole treatment. The patient is currently undergoing regular follow-up, and the recovery progress is reported to be favorable.

The majority of pulmonary echinococcosis cases are not characterized by distinctive imaging features, and the intricacy and diversity of their associated complications can lead to confusion with other pulmonary round lesions. These include, but are not limited to, specific types of lung cancer, congenital bronchogenic cysts, lung abscesses, pulmonary cysts, tuberculosis, and certain mediastinal tumors. Prior to surgery, the preoperative diagnosis for this case was a bronchogenic pulmonary cyst, with the possibility of pulmonary granular echinococcosis not being considered in the differential diagnosis. This oversight was primarily due to the fact that Yunnan Province is not recognized as a highly endemic area for echinococcosis within China, coupled with a modest level of experience in diagnosing and treating such cases. Consequently, in subsequent clinical investigations, patients with a documented history of pastoral exposure or consumption of uncooked meat or water, accompanied by imaging findings indicative of a rounded, uniform-density occupancy in the lungs, should be considered for the presence of fine-grained *Echinococcus granulosus* infection. Consequently, the implementation of ancillary diagnostic procedures, such as indirect hemagglutination tests or enzyme immunoassays for antibodies, along with the integration of molecular biology and advanced technologies, including polymerase chain reaction (PCR) and gene sequencing, will be warranted. These methodologies have the capacity to detect the DNA of *Echinococcus* tapeworm in tissue samples or body fluids, thereby facilitating a more precise diagnosis. In this particular instance, PCR testing was not performed due to an initial misdiagnosis. In future cases, the incorporation of molecular biology techniques should be contemplated when conditions allow to enhance diagnostic accuracy for precise treatment. Moreover, long-term follow-up and regular medication should be considered to assist treatment and prevent recurrence.

## Data Availability

The original contributions presented in the study are included in the article/supplementary material, further inquiries can be directed to the corresponding author.
